# Does inspiration of exhaled CO_2_ explain improved oxygenation with a face mask plus high-flow nasal cannula oxygen in severe COVID-19 infection?

**DOI:** 10.1186/s13054-021-03771-7

**Published:** 2021-09-20

**Authors:** Erik R. Swenson

**Affiliations:** Pulmonary and Critical Care Medicine, VA Puget Sound Health Care System, University of Washington, Seattle, WA 98108 USA

**Dear Sir,**

I read with interest the research letter by Dogani et al. [1] on the improvement in arterial oxygenation in spontaneously breathing patients with severe COVID-19 infection requiring high-flow nasal cannula oxygen (80% O_2_ at 40 l/min) with the application of a simple oxygen mask but with no further O_2_ added. The resultant striking rise in SpO_2_ from 90 to 95% in eighteen patients was associated with a non-significant 0.15 kPa (1.1 mmHg) rise in PaCO_2_ and a non-significant fall in respiratory rate of 2 breath/min. The authors ascribe these changes to some degree of elimination of ambient air entrainment by the mask such that the effective F_I_O_2_ was higher. While no argument can be made against this interpretation as a contributor, it is unlikely that with an already very high inspiratory flow rate of 40 l/min that minimization of ambient air entrainment with respiratory rates in the 20s fully explains the improvement. Another possibility is re-inspiration of exhaled CO_2_. This would be consistent with the slight rise in PaCO_2_. Addition of inspired CO_2_ improves regional ventilation-perfusion matching and oxygenation with unchanged minute ventilation and tidal volume [2] by its several actions on pulmonary vascular resistance, airways resistance and parenchymal compliance [3]. Additionally, even the slight rise in PaCO_2_ stimulates ventilation by increasing tidal volume which will reduce V_D_/V_T_ and dead space ventilation by decreasing the fraction of the inspired volume needed to clear the anatomic (conducting airways) dead space. Whatever the explanation(s) the finding, if verified in other studies, is clinically important. Adding a mask is easy to administer and in situations of oxygen scarcity, a simple means to reduce use of a limited resource.

## Data Availability

Not applicable to a letter to the editor in response to a published paper.
